# Effects of isometric resistance training and detraining on ambulatory blood pressure and morning blood pressure surge in young normotensives

**DOI:** 10.3389/fphys.2022.958135

**Published:** 2022-09-09

**Authors:** A. W. Baross, A. D. Kay, B. A. Baxter, B. H. Wright, C. L. McGowan, I. L. Swaine

**Affiliations:** ^1^ Sport and Exercise Science, University of Northampton, Northampton, United Kingdom; ^2^ Department of Kinesiology, University of Windsor, Windsor, ON, Canada; ^3^ Sport Science, University of Greenwich, London, United Kingdom

**Keywords:** hypertention, detraining effect, isometric resistance training, ambulatory, blood pressure

## Abstract

Isometric resistance training (IRT) has been shown to reduce resting and ambulatory blood pressure (BP), as well as BP variability and morning BP surge (MBPS). However, there are no data available regarding how long after cessation of IRT these effects are maintained. Therefore, the purpose of this study was to determine the effects of 8 weeks of detraining on resting BP, ambulatory BP and MBPS following 8 weeks of IRT in a population of young normotensive individuals and to further substantiate previously reported reductions in MBPS following IRT. Twenty-five apparently healthy participants with resting BP within the normal range (16 men, age = 23 ± 6 years; 9 women, age = 22 ± 4 years, resting BP: 123 ± 5/69 ± 7 mmHg) were randomly assigned to a training-detraining (TRA-DT, *n* = 13) or control (CON, *n* = 12) group. Resting BP, ambulatory BP and MBPS were measured prior to, after 8 weeks of bilateral leg IRT using an isokinetic dynamometer (4 × 2-min contractions at 20% MVC with 2-min rest periods, 3 days/week) and following an 8-week detraining period. There were significant reductions in 24-h ambulatory systolic BP (SBP) and calculated SBP average real variability (ARV) following IRT that were maintained after detraining (pre-to-post detraining, −6 ± 4 mmHg, *p* = 0.008, −2 ± 1.5 mmHg, *p* = 0.001). Similarly, the training-induced decreases in daytime SBP and daytime SBP ARV (pre-to-post detraining, −5 ± 6 mmHg, *p* = 0.001; −2 ± 1.2 mmHg, *p* = 0.001, respectively), MBPS (pre-to-post detraining, −6 ± 9 mmHg, *p* = 0.046) and resting SBP (pre-to-post detraining, −4 ± 6 mmHg, *p* = 0.044) were preserved. There were no changes in night-time or night-time SBP ARV across all time points (pre-to-post detraining, −1 ± 8 mmHg, *p* = 1.00, −0.7 ± 2.9 mmHg, *p* = 1.00). These results confirm that IRT causes significant reductions in resting BP, ambulatory BP, ambulatory ARV and MBPS. Importantly, the changes remained significantly lower than baseline for 8 weeks after cessation of training, suggesting a sustained effect of IRT.

## 1 Introduction

The World Health Organisation (World Health Organization, 2013) have reported hypertension, a major risk factor for cardiovascular disease (CVD), stroke and coronary heart disease (CHD), to be near epidemic levels globally. Further, it has been identified as one of their globally targeted non-communicable disease risk factors (WHO, 2016). Additionally, the within-day variations in twenty-four hour (24-h) ambulatory BP are thought to be strongly associated with the risk of cardiovascular events and stroke (Kario et al., 2003). One normal occurrence associated with the diurnal variation in BP is the surge in morning BP seen upon waking and during early morning (6–10 a.m.), which is linked to end organ damage and considered to be a destabilising factor for atherosclerotic plaques (White, 2001; Kario, 2005; Kario, 2010). Morning BP surge (MBPS) occurs upon waking and is a result of the drop in nocturnal BP and the subsequent rise in BP on awakening. Elevated MBPS (∼4–6 h upon wakening) is a significant risk factor for CVD (Kario et al., 2003; Dolan et al., 2008; Li et al., 2010) and may be responsible for the increased frequency of early morning cardiovascular events (Kario, 2010).

Isometric resistance training (IRT) using either a lower body (leg extension or Squats) or handgrip exercise has been established as one of the best forms of nonpharmacological interventions for the prevention and treatment of hypertension and endorsed by the American College of Cardiology/American Heart Association (Whelton and Carey, 2017), in addition to being included in the recent Australian position stand on exercise and hypertension (Sharman et al., 2019). IRT programmes undertaken thrice weekly (4 × 2-min isometric contractions) at 20%–30% of an individual’s maximum voluntary contraction (MVC) for a period of up to 10 weeks have demonstrated reductions in 24-h ambulatory (−4 mmHg), daytime (−3 to −6 mmHg) and night-time (−3 to −4 mmHg) BP in normotensives, with no reported difference in the magnitude of these reductions between women and men (Somani et al., 2017; Baross et al., 2022).

Despite the weight of evidence that supports the health benefits of exercise, particularly IRT (Carlson et al., 2014; Inder et al., 2016; Smart et al., 2019; Edwards et al., 2022), it is still a challenge for individuals to maintain regular physical activity or structured exercise programmes (Bonsu and Terblanche, 2016). For a large proportion of those that do engage, interruptions or the cessation in training (termed detraining) due to unforeseen circumstances, such as illness or injury are likely to occur (Volaklis et al., 2006). Therefore, to fully determine the efficacy of a IRT programme it is important to quantify the changes associated with an often-prolonged period of detraining.

Reports suggest that reductions in BP following aerobic and resistance training can return to pre-training levels within as little as 2 weeks following a detraining period (Meredith et al., 1990; Murray et al., 2006; Moker et al., 2014). Although previous studies have evaluated the effects of detraining following aerobic and resistance (Elliott et al., 2002; Fatouros et al., 2006; Moker et al., 2014) exercise periods, few studies (Wiley et al., 1992; Howden et al., 2002) have reported the effects of detraining following IRT in young healthy individuals. Since interruptions in training or prescribed exercise programmes are likely to occur it is important to quantify changes in ambulatory BP resulting from the cessation of exercise. To the authors’ knowledge, there is little published research that quantifies the changes in BP that occur as a result of the cessation of an IRT programme in young healthy adults, particularly 24-h ambulatory BP. Both Wiley et al. (1992) and Howden et al. (2002) suggest resting BP returns to pre-training levels following a period (5–8 weeks) of detraining. Therefore, it is important to not only establish the time scale of the BP reductions but to also know how rapidly they regress following the cessation of the training. The latency and the continued regression in the adaptations associated with the reduced BP are particularly significant for individuals at risk of CVD.

Therefore, the aim of this work was to 1) determine the effect of detraining on ambulatory BP and MBPS in young normotensive men and women following an 8-week IRT programme and an 8-week detraining period and 2) to substantiate previously reported reductions in MBPS following IRT.

## 2 Materials and methods

### 2.1 Participants

Twenty five (Males; *n* = 16; mean ± SD: age 23 ± 4 years, height 175 ± 8 cm, mass 78 ± 8 Kg; Females; *n* = 9; mean ± SD: age 23 ± 6 years, height 163 ± 8 cm, mass 67 ± 6 Kg) normotensive (123 ± 5/69 ±7 mmHg), recreationally active (IPAQ) but not resistance trained individuals were recruited and randomly assigned (using an online random numbers generator) to either a training-detraining (TG-DT; *n* = 13; age 23 ± 4 years, height 171 ± 4 cm, mass 72 ± 8 Kg) or a Control (CON; *n* = 12; age 22 ± 4 years, height 171 ± 10 cm, mass 74 ± 8 Kg) group. Participants undertook between 2–4 moderate intensity exercise sessions (on average 30–45 min duration) per week, which they continued to perform during the 8-week IRT period. They did not engage in smoking or vaping (Bolinder et al., 1998; Crippa et al., 2018) and were not prescribed medication. After receiving institutional ethical approval from the University of Northampton’s Ethics review panel all participants received a detailed information sheet explaining the experimental protocol and potential risks involved, then completed and signed a pre-test medical questionnaire and informed consent form.

**FIGURE 1:**
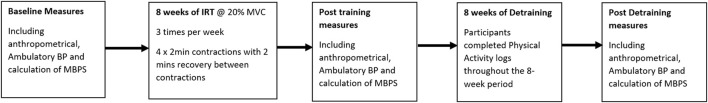
Study flow diagram illustrating the Isometric resistance training (IRT) and detraining periods and the points of blood pressure measures.

Following a familiarisation session, baseline data collection was completed. Participants then, individually undertook 8 weeks of supervised bilateral isometric leg extension (ILE) training. Post-intervention testing (mid-point) took place following the completion of the training programme within 48–72 h of the final training session and within 2 h of baseline data collection time. The detraining period started on the first week following the final training session and continued for 8 weeks, with post detraining measures (end-point) taken at the end of the final week of detraining (see Figure 1). All data collection sessions were undertaken by the same individual in a temperature-controlled environment (20–23°C) at least 2 hours post-prandial. Participants abstained from caffeine in the 12 h prior to testing and did not take over the counter medication, undertake vigorous exercise or consume alcohol for 24 h preceding the data collection sessions. All data collection measures in women were tested in their early follicular phase (days 1–7 of their menstrual cycle; *n* = 3) (Tsai et al., 2003) or during placebo pill ingestion for women taking oral contraceptives (*n* = 6; 11, 28). Previous research has noted no significant difference in BP changes between males and females following IRT (Somani et al., 2017; Baross et al., 2022). All procedures were conducted with the intent to treat.

### 2.2 Protocol

#### 2.2.1 Resting blood pressure and heart rate

Resting systolic (SBP), diastolic (DBP) blood pressure, mean arterial pressure (MAP) and heart rate (HR) were recorded using an automatic blood pressure monitor (UA-767 Plus, A&D Company, Ltd., Tokyo, Japan) and a HR monitor (Polar Beat, Polar Electro, Kempele, Finland). Three measurements were taken following a 10-min rest in a supine position, at 1-min intervals with the lowest value used to determine resting HR and BP (Pickering et al., 2005).

#### 2.2.2 Ambulatory blood pressure

24-h, daytime and night-time ambulatory BP was measured using a portable BP monitor (P.M.S. Instruments Ltd. Maidenhead, Berks, United Kingdom). Daytime measures, defined as the time participants rose until they retired to bed were recorded at 30-min intervals, whilst night-time measures, defined as the time participants went to bed until they awoke were recorded hourly (Amici et al., 2009; Verdecchia et al., 2012). 24-h ambulatory BP recordings were discarded when there was a missing hour of data or < 80% valid measures were present (Gosse et al., 2004; Hernández-del Rey et al., 2007). To help standardise BP measures, participants used a physical activity log to report the activities undertaken during the baseline ambulatory BP monitoring period, including their sleep times, any changes in diet and were subsequently asked to undertake similar activities and sleep times for the ambulatory BP measure post training and following the detraining period (O’Brien et al., 2013; Parati et al., 2014), again monitored using the physical activity log. During the detraining period participants were instructed to maintain the same physical activity levels undertaken and recorded weekly, during the 8-week IRT period without the additional IRT, in an attempt to standardise the physical activity levels during the detraining period. Again, physical activity was recorded weekly during the detraining period.

#### 2.2.3 Morning blood pressure surge and average real variability calculations

MBPS was calculated as previously reported (Carlson et al., 2014) using the mean of the four SBP readings in the 2-h period just after waking minus the mean of the two readings centred around the lowest nocturnal SBP reading, but not adjusted for 24-h ambulatory BP (Amici et al., 2009; Verdecchia et al., 2012). Average real variability (ARV) of ambulatory (24-h, Daytime and Night-time) SBP and DBP, a measures of BP variability were calculated as previously reported (Boardman et al., 2017). Participant baseline data including mean values for the morning SBP surge are presented in Table 1.

**TABLE 1:** Participant baseline demographic and ambulatory data.

	TG-DT group (*n* = 13)	CON group (*n* = 12)
Age (yrs)	23 ± 4	22 ± 4
Height (cm)	171 ± 9	171 ± 10
Body mass (kg)	72 ± 8	74 ± 8
Resting heart rate	68 ± 11	69 ± 9
Resting BP		
SBP (mmHg)	123 ± 5	123 ± 6
DBP (mmHg)	70 ± 8	69 ± 6
MAP (mmHg)	88 ± 6	87 ± 6
Ambulatory BP		
24-h SBP (mmHg)	123 ± 5	123 ± 5
24-h DBP (mmHg)	63 ± 7	65 ± 3
Daytime SBP (mmHg)	125 ± 7	126 ± 5
Daytime DBP (mmHg)	67 ± 8	68 ± 6
Night-time SBP (mmHg)	110 ± 8	107 ± 5
Night-time DBP (mmHg)	55 ± 8	57 ± 4
Morning SBP (mmHg)	124 ± 7	119 ± 5
Lowest night-time SBP (mmHg)	102 ± 4	99 ± 4
MBPS (mmHg)	22 ± 7	20 ± 6

Values are means ± SD. TG-DT, detraining group; CON, control group; SBP, systolic blood pressure; DBP, diastolic blood pressure; MAP, mean arterial pressure; MBPS, morning blood pressure surge.

#### 2.2.4 Isometric resistance training

Participants completed 8 weeks of supervised, onsite IRT three times per week (separated by at least 24 h) using an isokinetic dynamometer (Biodex Medical Systems Inc. New York, United States). Training consisted of 4 × 2-min isometric contractions at 20% MVC (reassessed at the end of week 2, 4 and 6), with 2-min rest periods between contractions. MVC was determined as the highest value following three, 2–4 s maximum bilateral knee extension (Baross et al., 2013). Additionally, dietary, nutritional and exercise changes were monitored and recorded throughout both the 8-week training programme and the 8-week detraining period using personal physical activity logs.

### 2.3 Statistical analysis

Data were assessed for normal distribution and satisfied parametric assumptions (Field, 2005). Statistical analysis was performed using IBM SPSS Statistics 26 software (SPSS Inc., Chicago, Illinois, United States). A two-way repeated measures ANOVA was used to determine the significant difference within and between groups for ambulatory (mean 24-h, daytime, night-time and diurnal variation) BP and MBPS at baseline compared to post training (mid-point) and detraining (end-point). Post hoc analysis (Bonferroni) was used to further assess specific significant differences. Statistical significance was set at *p* ≤ 0.05. All data are presented as mean ± SD.

The day-to-day reliability of the ambulatory BP measures was determined by calculating the intraclass correlation coefficient (ICC) and coefficient of variation (expressed as a percentage on the mean). For 24-h SBP, daytime SBP and night-time SBP no significant difference was detected between day-to-day mean values for any measure. The ICC values were 0.73, 0.71 and 0.81 and the coefficients of variation were 2.5%, 2.3%, and 2.6%, respectively.

### 2.4 Sample size

Based on previous studies the effect size (Cohen’s *d*) was calculated from mean changes in ambulatory blood pressure [24 h SBP (ES = 1.33), Daytime SBP (ES = 1.38), MBPS (ES = 1.40)] using previous studies employing similar interventions (Somani et al., 2017; Baross et al., 2022). To ensure adequate statistical power for all analyses, power analysis was conducted for 24 h SBP (i.e., the variable with the smallest effect size) using the following parameters (variable = 24 h SBP, power = 0.80, alpha = 0.05, effect size = 1.33). The analysis revealed that the total sample size required for statistical power was 20, therefore, accounting for 20% attrition rates a minimum of 24 subjects were initially recruited.

## 3 Results

Participants completed ≥ 95% of the twenty-four training sessions over the 8-week IRT programme. Baseline data (Table 1) showed no significant difference (*p* > 0.05 in all cases) between TG-DT and CON group for age, height, body mass, and all BP measures. Throughout the 16-week training and detraining periods participants reported no changes in their exercise routine or diet. Additionally, no adverse effects of the training intervention or the data collection sessions were reported.

### 3.1 Effects of isometric resistance training on resting blood pressure

**FIGURE 2:**
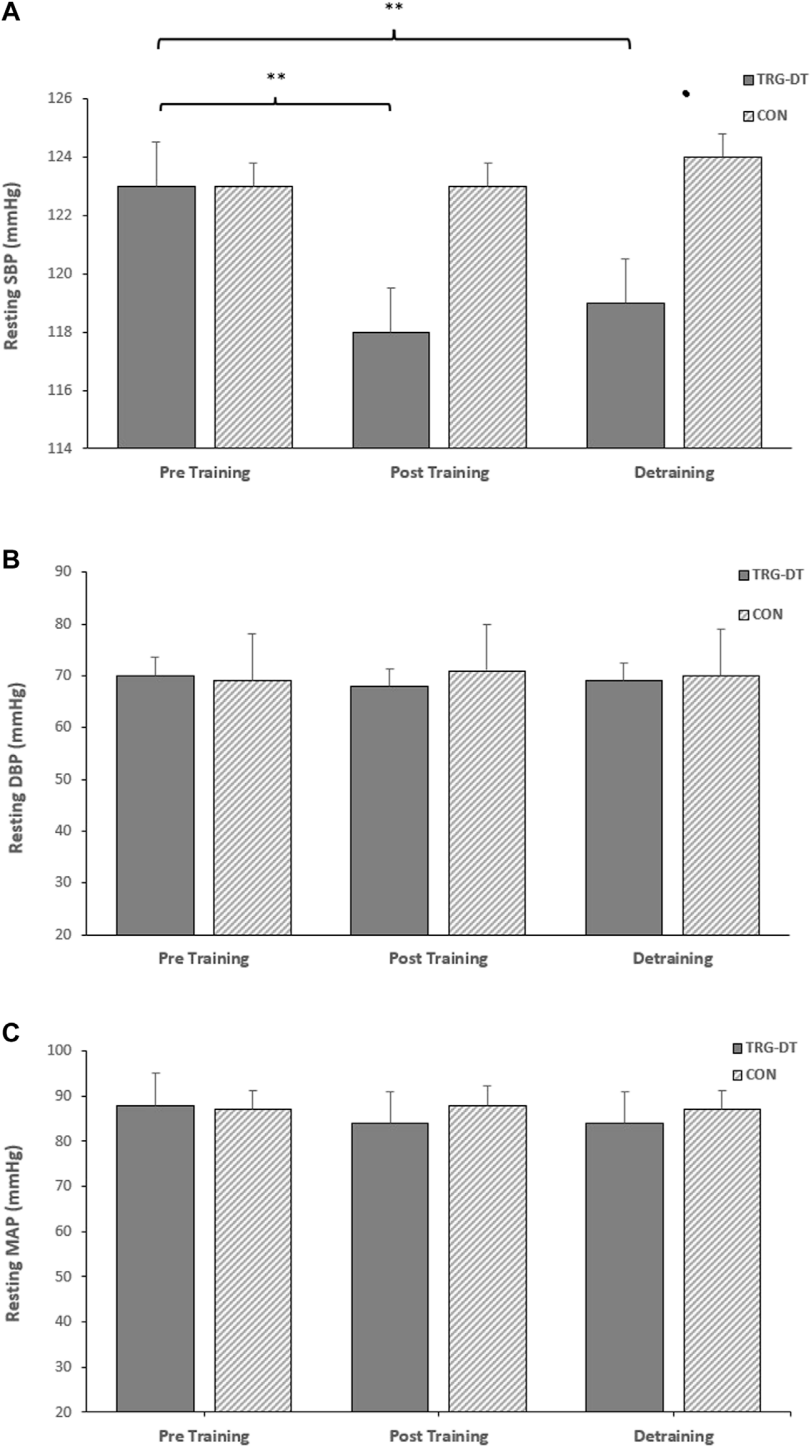
Effects of 8 weeks of isometric resistance training (IRT) followed by 8 weeks of detraining on **(A)**, resting systolic blood pressure (SBP) **(B)**, resting diastolic blood pressure (DBP) and **(C)**, resting mean arterial pressure (MAP) for isometric training-detraining (TRG-DT) and control (CON) groups. *p value < 0.005, **p value < 0.01.

There were significant reductions in resting SBP after the completion of the 8-week IRT training period (−6 ± 6 mmHg, *p* = 0.005). However, there were no significant changes in the remaining resting measures (DBP, −2 ± 8 mmHg, MAP, −3 ± 6 mmHg, *p* > 0.05 in both cases). There were no significant differences in the CON group’s resting BP (SBP, DBP and MAP; *p* > 0.05 in all cases, see Figure 2).

### 3.2 Effects of isometric resistance training on ambulatory blood pressure, average real variability and morning BP surge

**FIGURE 3:**
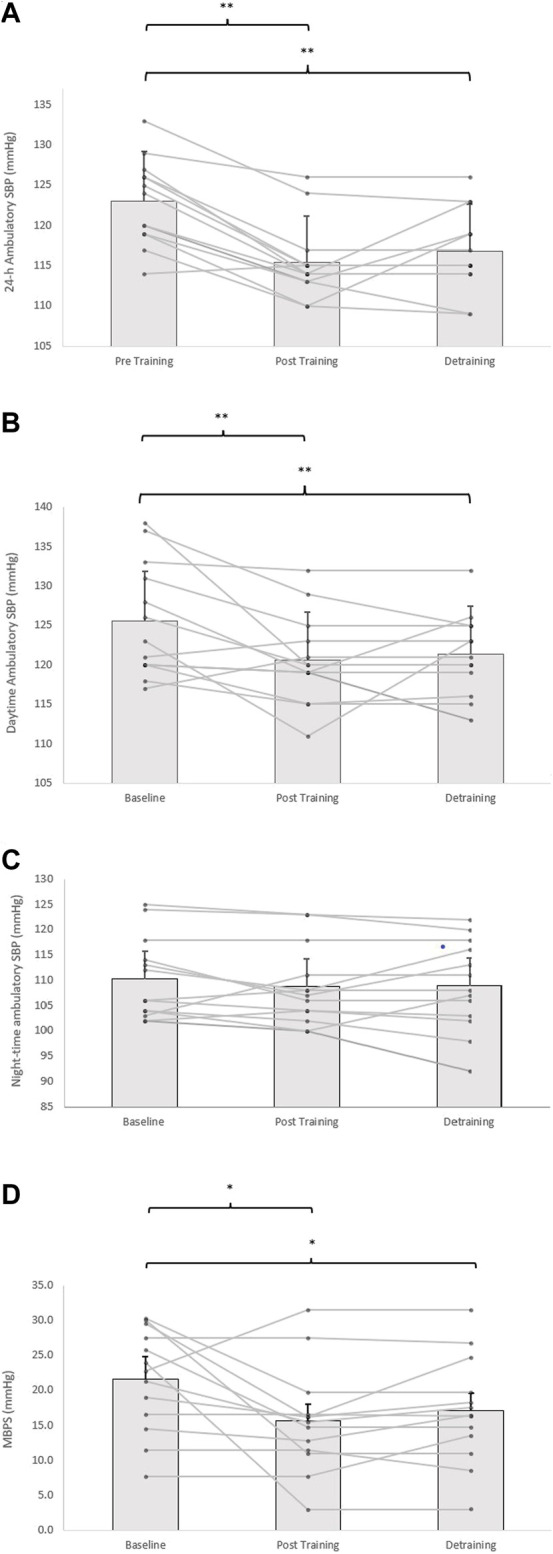
Effects of 8 weeks of isometric resistance training (IRT) followed by 8 weeks of detraining for individual participants and mean data on **(A)**, 24-h **(B)**, daytime **(C)**, Night-time ambulatory systolic blood pressure (SBP) and **(D)**, morning blood pressure surge (MBPS) for isometric training-detraining (TRG-DT). *p value < 0.005, **p value < 0.01.

**FIGURE 4:**
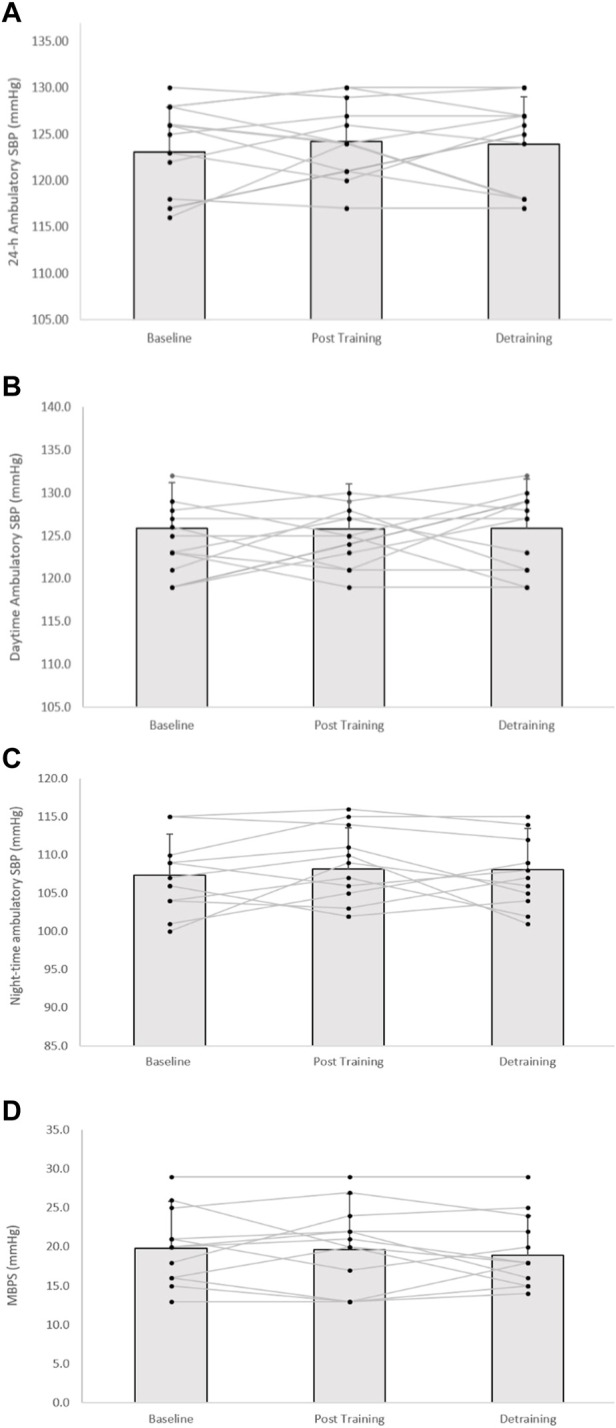
Effects of 8 weeks of isometric resistance training (IRT) followed by 8 weeks of detraining for individual participants and mean data on **(A)**, 24-h **(B)**, daytime **(C)**, Night-time ambulatory systolic blood pressure (SBP) and **(D)**, morning blood pressure surge (MBPS) for control (CON) groups. *p value < 0.005, **p value < 0.01.

Following the 8-week IRT programme there were significant reductions in 24-h ambulatory SBP (−8 ± 4 mmHg, *p* = 0.001) and daytime SBP (−5 ± 6 mmHg, *p* = 0.001), alongside 24-h and daytime SBP ambulatory ARV (−2.16 ± 1.37 mmHg, *p* = 0.001; −1.85 ± 1.21 mmHg, *p* = 0.001, respectively). In addition, analysis of the calculated MBPS demonstrated significant reductions (−6 ± 9 mmHg, *p* = 0.042) within the TG-DT group. However, there was no significant change in night-time SPB (−1 ± 8 mmHg, *p* = 1.000) or night-time SBP ARV (−0.66 ± 2.93 mmHg, *p* = 1.000). Despite some observed changes in ambulatory DBP (24-h, 63 ± 7 to 62 ± 7 mmHg; daytime, 67 ± 8 to 66 ± 5 mmHg; night-time, 55 ± 8 to 55 ± 7 mmHg) within the TG-DT group, over time these changes were not significant (*p* > 0.05 in all cases). Nor were there any changes in 24-h, daytime and night-time DBP ARV (*p* > 0.05 in all cases). Furthermore, there were no significant differences in the CON group’s ambulatory BP, ARV (24-h, daytime, night-time) and calculated MBPS over the same period (*p* > 0.05 in all cases, see Figures 3, 4; Table 2).

**TABLE 2:** Ambulatory SBP ARV results at baseline, post 8-week IRT and post 8-week detraining TRG-DT control.

Ambulatory SBP ARV (mmHg)	Baseline	Post 8-week IRT	Post 8-week detraining	Baseline	Post 8-week IRT	Post 8-week detraining
24-h	10.21 ± 1.88	8.05 ± 1.47**	8.33 ± 1.23**	10.37 ± 2.23	10.50 ± 2.01	10.30 ± 2.32
Daytime	10.84 ± 2.17	8.99 ± 1.67**	9.03 ± 1.50**	10.54 ± 2.09	10.69 ± 2.06	10.46 ± 2.32
Night-time	7.92 ± 3.24	7.27 ± 1.98	7.50 ± 2.27	7.08 ± 2.13	7.37 ± 2.23	7.87 ± 2.61

Data are presented as mean ± SD. (TRg-DT group *n* = 13; Control group *n* = 12). SBP, systolic blood pressure; ARV, average real variability. *p* values represent changes within groups over time compared to baseline measures. **p value < 0.01.

### 3.3 Effects of detraining on resting blood pressure

Within the TG-DT group there was no change or a small but non-significant increase in resting SBP, DBP and MAP following the completion of the IRT and the end of the 8-week detraining period (SBP; 117 ± 6 to 119 ± 4 mmHg; DBP; 68 ± 9 to 68 ± 8 mmHg; MAP; 84 ± 6 to 84 ± 5 mmHg; *p* > 0.05 in all cases). Additionally, the reported significant reductions in baseline SBP following 8 weeks of IRT were maintained following the detraining period (−4 ± 6 mmHg, *p* = 0.044), with no significant changes in the remaining resting measures (DBP, −1 ± 6 mmHg; MAP, −3 ± 5 mmHg; see Figure 2).

### 3.4 Effects of detraining period on ambulatory blood pressure, average real variability and morning BP surge

Although there was a slight rise in both ambulatory SBP and ARV (24-h, daytime) and MBPS from the cessation of training to the completion of the 8-week detraining period (24-h; 115 ± 5 to 117 ± 5 mmHg; daytime; 121 ± 6 to 121 ± 5 mmHg; ARV; 24-h; 8.05 ± 1.47 to 8.33 ± 1.23 mmHg; daytime; 8.99 ± 1.67 to 9.03 ± 1.50 mmHg; MPBS; 16 ± 8 to 16 ± 7 mmHg, respectively, *p* > 0.05 in all cases), significant reductions within the TG-DT group were maintained from baseline measures in 24-h ambulatory (−6 ± 4 mmHg, *p* = 0.008), daytime (−5 ± 6 mmHg, *p* = 0.001) SBP, 24-h SBP ARV (−1.88 ± 1.52 mmHg, *p* = 0.002), daytime SBP ARV (−1.81 ± 1.35 mmHg, *p* = 0.001) and MBPS (−6 ± 9 mmHg, *p* = 0.046, see Figure 2). There were no significant changes in night-time SPB (−1 ± 6 mmHg, *p* = 1.00) or night-time SBP ARV (−0.43 ± 2.87 mmHg, *p* = 1.00). Additionally, there were no significant changes in ambulatory DBP (24-h, 62 ± 7 to 65 ± 6 mmHg; daytime, 66 ± 5 to 68 ± 7; night-time, 55 ± 7 to 55 ± 9, *p* > 0.05 in all cases) or DBP ARV (24-h, 7.10 ± 2.75 to 7.47 ± 2.65 mmHg; daytime, 8.19 ± 2.73 to 8.32 ± 1.99; night-time, 8.03 ± 3.28 to 7.82 ± 2.78, *p* > 0.05 in all cases) within the TG-DT group, over time.

### 3.5 Between group ambulatory blood pressure, average real variability and morning BP surge analysis

**FIGURE 5:**
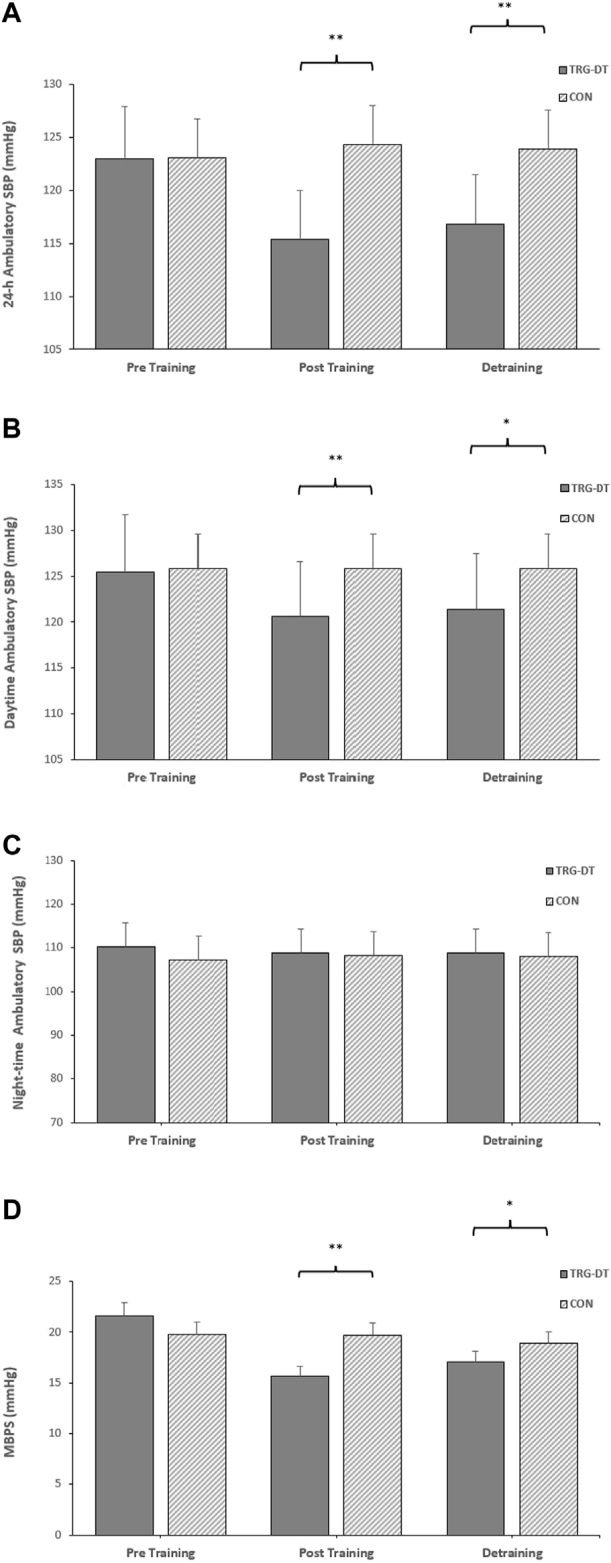
The between-groups effects of 8 weeks of isometric resistance training (IRT) followed by 8 weeks of detraining on **(A)**, 24-h **(B)**, daytime **(C)**, Night-time ambulatory systolic blood pressure (SBP) and **(D)**, morning blood pressure surge (MBPS) for control (CON) groups. *p value < 0.005, **p value < 0.01.

At the completion of the 8-week training period there were significant differences in 24-h ambulatory SBP (115 ± 5 to 124 ± 5 mmHg, *p* = 0.001), daytime SBP (121 ± 6 to 126 ± 5 mmHg, *p* = 0.016), 24-h SBP ARV (8.05 ± 1.47 to 10.50 ± 2.01 mmHg, *p* = 0.004), daytime SBP ARV (8.99 ± 1.67 to 10.84 ± 2.17 mmHg, *p* = 0.037) and MPBS (16 ± 8 to 20 ± 7 mmHg, *p* = 0.02) between the TG-DT and the CON group with no corresponding significant differences in night-time SBP (109 ± 8 to 108 ± 6 mmHg, *p* = 0.598) or night-time SBP ARV (7.42 ± 1.98 to 7.08 ± 2.13 mmHg, *p* = 0.337). Between group differences were maintained following the detraining period (24-h, 117 ± 5–124 ± 5 mmHg, *p* = 0.02; daytime, 121 ± 5–127 ± 7 mmHg, *p* = 0.042; 24-h SBP ARV, 8.33 ± 1.23–10.30 ± 2.32 mmHg, *p* = 0.015; daytime SBP ARV, 9.03 ± 1.50–10.46 ± 2.32 mmHg, *p* = 0.034; MBPS, 16 ± 7 to 21 ± 5 mmHg, *p* = 0.049; see Figure 4). The data analysis confirmed that there were no significant differences in ambulatory DBP or DBP ARV at the end of the IRT programme (mid-point) or following the detraining period (*p* > 0.05 in both cases; see Figure 5; Table 2).

## 4 Discussion

This study is the first to investigate the effects of a similar period of detraining on resting and 24-h ambulatory BP following an 8-week IRT intervention in young normotensive men and women. Furthermore, the data from the initial 8-week IRT period is in agreement with previous studies reporting significant reductions in ambulatory BP (Somani et al., 2017; Baross et al., 2022) and MBPS (Baross et al., 2022) following training for both men and women. Importantly, the novel data indicates that the reductions in resting and ambulatory BP seen after the 8-week IRT intervention in young normotensives are sustainable over an 8-week detraining period. Indeed, a significant difference in both resting and ambulatory BP (24-h SBP, daytime SBP and MBPS) compared to baseline values were still evident after the 8 weeks of detraining.

### 4.1 Effects of isometric resistance training

There is a reported (World Health Organization, 2013) global rise in non-communicable diseases including hypertension, a key risk factor for CVD. There also appears to be a closer association between CVD and changes in ambulatory BP than the more commonly used clinical BP, particularly diurnal variations such as MBPS (Kario, 2010) and the average real variation in ambulatory blood pressure (Stevens et al., 2016; Boardman et al., 2017). Therefore, the reported changes in ambulatory BP and more specifically the significant decrease in MBPS, which support previous findings (Baross et al., 2022) and the reported reductions in 24-h and daytime SBP ARV further substantiate the reported benefits of IRT for the chronic management of BP in young healthy individuals (Wiles et al., 2010; Somani et al., 2017; Baross et al., 2022). Further, as exercise programmes tend to be more effective at lowering BP in hypertensive populations it is likely to also be effective in borderline- and hypertensive populations (Cornelissen and Smart, 2013).

The significant reductions in ambulatory BP, including 24-h (−8 mmHg) and daytime ambulatory BP (−5 mmHg) reported in the present study following the initial 8-week training period are similar to those reported in previous IRT normotensive studies, 24-h, (−4 mmHg) and daytime ambulatory BP, −3 to −5 mmHg; (Somani et al., 2017; Baross et al., 2022). The lack of significant changes in night-time ambulatory BP are also comparable to those reported recently (Baross et al., 2022) but differ from other studies (Stiller-Moldovan et al., 2012; Somani et al., 2017). Further, the significant reductions in MBPS (−6 mmHg) in the present study confirm the previously reported novel MBPS data (−6 mmHg; 12). The inclusion of a control group in this study, a reported limitation of the Baross et al. (2022) research substantiates the between-group data, indicating that in addition to the reported significant reduction in MBPS over time, there was a significant difference in MBPS between the training and control group post 8-week IRT. Collectively, this adds further weight to the previously published MBPS literature. Additionally, the reductions in 24-h SBP ARV (2.16 mmHg) are similar to those reported by Taylor et al. (2019) although their study used unmedicated hypertensives. The significant reductions in resting SBP data reported here (−6 mmHg), is again comparable with previous IRT studies (−5 to −11 mmHg) as are the small but, in this case, non-significant reductions in DBP and MAP (McGowan et al., 2007; Wiles et al., 2010; Baross et al., 2012; Smart et al., 2019).

### 4.2 Effects of detraining

Despite the associated health benefits of exercise programmes on hypertension and CVD (World Health Organization, 2013; Bonsu and Terblanche, 2016; Whelton and Carey, 2017) the engagement in regular therapeutic exercise is still a challenge for most individuals (Bonsu and Terblanche, 2016). Interestingly, this study’s novel findings show that although there was a slight increase in resting SBP following the 8-week detraining period (+2 mmHg) there remained a significant reduction compared to pre-training levels (−4 mmHg); this trend is also evident in the reported ambulatory BP measures. Twenty 4 h ambulatory SBP (+2 mmHg), 24-h SBP ARV (+0.28 mmHg), and MBPS (+1 mmHg) were seen to increase slightly from post-training levels but remained significantly lower than the reported pre-training levels (24-h, −6 mmHg; 24-h SBP ARV −1.88 mmHg; MBPS, −6 mmHg). Similarly, the reductions seen in daytime ambulatory SBP at the end of the IRT were maintained over the course of the detraining period (−5 mmHg), as was daytime SBP ARV (−1.81 mmHg).

Moraes et al. (2012) reported similar trends to the present study using conventional resistance training, which reported no regression post-detraining effects for resting SBP and DBP following a 4-week detraining period, preceded by a 12-week training programme. However, other studies have reported a regression to, or close to, baseline values following various lengths of detraining preceded by a period of resistance training (Moker et al., 2014) or a combination of resistance and aerobic training (Volaklis et al., 2006; Moker et al., 2014). In addition, Howden et al. (2002) reported no difference from baseline resting SBP measures to those taken after 5 weeks of isometric leg training followed by an 8-week washout period (in preparation for another isometric training period), in effect a detraining period. However, the study’s focus was not to investigate the effects of the detraining period and therefore the effects of the washout period were not fully reported.

More specifically, the data differs from the only previous IRT study to investigate the effects of a detraining period (Wiley et al., 1992) which reported a rapid regression in the resting SBP and DBP over a 5-week detraining period following an initial 5-week training period. The disparity between Wiley et al. (1992) and the present study may be due, in part to the different methodologies (including mode, intensity, duration and frequency of exercise) as well as differences in the outcome measure (previous study only reported resting BP). Furthermore, it is thought that the length of the training intervention may impact on the mechanisms responsible for the reported decrease in BP (Tinken et al., 2008; Baross et al., 2012) in that, the vascular adaptations appear to be biphasic. It has been suggested that IRT of 4 weeks or less is predominately associated with functional adaptations, whilst training periods of 8 weeks or more are thought to elicit structural adaptations, such that the vasculature is remodelled in an attempt to normalise the elevated shear stress (Tinken et al., 2008; Baross et al., 2012). Therefore, differences in vascular mechanisms associated with the exercise induced reductions in BP could be responsible for the difference in the time course of the BP responses following the detraining periods and therefore, in part explain the difference in the rate of BP regression between the present study and Wiley et al. (1992).

However, others (Verdecchia et al., 2012; Thomas, 2015; Mortensen et al., 2019) have suggested additional mechanisms that may be responsible for the reported reductions in both resting and ambulatory BP. These include altered endothelial function, either through improved endothelial cell turnover (Murray et al., 2006) or increased release of endothelial derived dilatory factors such as nitric oxide (NO) or NO-synthase (Wilkinson et al., 2004; Murray et al., 2006). In addition, Baross et al. (Carlson et al., 2014) discussed the role of functional sympatholysis as a possible mechanism for the reported reductions in BP following IRT (Verdecchia et al., 2012; Thomas, 2015; Mortensen et al., 2019). Further, we are not aware of any published research that has specifically investigated whether the mechanisms associated with the eventual rise in BP following an extended detraining period mirror to those associated with the reduced BP following IRT.

### 4.3 Practical implications

The presented data demonstrates that the BP lowering effects of IRT have remained sustained for the length of the 8-week detraining period such that, resting, 24-h SBP, daytime ambulatory SBP and MBPS remained significantly lower than the observed BP values prior to the commencement of the IRT programme. The findings of the present study highlight the potential clinical benefits of this training programme and may help to establish the role of IRT in the prescription of exercise for the treatment of hypertension. Further, since interruptions in training are likely to occur in individuals undertaking prescribed exercise it is important to quantify the length of detraining where the clinical benefits remain. Finally, there is still limited data on the amount of IRT needed each week once the required BP reductions have been achieved. With adherence to prescribed exercise still a challenge, the possibility of having a reduced weekly maintenance dose to maintain the clinical benefits of IRT may be embraced by individuals undertaking these exercise programmes.

## 5 Conclusion

The most important observation of the present study was that the significant reductions in resting SBP, ambulatory BP (24-h SBP, daytime SBP) and MBPS seen following 8 weeks of IRT remained significantly reduced after 8 weeks of detraining. The results further support the previous resting and ambulatory BP research showing IRT to be an effective intervention for reducing BP. The findings also confirm the recently reported reductions in MBPS following an IRT programme in normotensive individuals. These novel findings have important clinical implications and would indicate that IRT may be beneficial for the chronic management of BP in young healthy individuals. One limitation of the study is the recruitment of normotensive male and female participants whose physiology differ from that of borderline-hypertensive or hypertensive populations. However, it was felt that it would be advisable to investigate the possible detraining effects in a healthy normotensive population initially. Further, both resting and ambulatory BP was only measured at the end of the detraining period, in future studies it may be advantageous to undertake these measures periodically during the detraining period to get a better understanding of the trend of any possible BP regression.

## Data Availability

The raw data supporting the conclusion of this article will be made available by the authors, without undue reservation.
